# Association of Gut *Lachnospiraceae* and Chronic Spontaneous Urticaria

**DOI:** 10.3390/life13061280

**Published:** 2023-05-30

**Authors:** Diana Ćesić, Liborija Lugović Mihić, Petar Ozretić, Ivana Lojkić, Marija Buljan, Mirna Šitum, Mario Zovak, Dinko Vidović, August Mijić, Nada Galić, Arjana Tambić Andrašević

**Affiliations:** 1 Department of Dermatology and Venereology, Medikol Clinic, 10000 Zagreb, Croatia; 2 School of Dental Medicine, University of Zagreb, 10000 Zagreb, Croatia; liborija@sfzg.hr (L.L.M.); buljan.marija@gmail.com (M.B.); mirna.situm@kbcsm.hr (M.Š.); mario.zovak@kbcsm.hr (M.Z.); dinko.vidovic@kbcsm.hr (D.V.); august.mijic@kbcsm.hr (A.M.); nada.galic@sfzg.hr (N.G.); arjana.tambic@bfm.hr (A.T.A.); 3 Department of Dermatology and Venereology, Sestre milosrdnice University Hospital Centre, 10000 Zagreb, Croatia; 4 Laboratory for Hereditary Cancer, Division of Molecular Medicine, Ruđer Bošković Institute, 10000 Zagreb, Croatia; pozretic@irb.hr; 5 Croatian Veterinary Institute, 10000 Zagreb, Croatia; ilojkic@veinst.hr; 6 Department of Surgery, Sestre milosrdnice University Hospital Centre, 10000 Zagreb, Croatia; 7 Department of Clinical Microbiology, University Hospital for Infectious Diseases, 10000 Zagreb, Croatia

**Keywords:** chronic spontaneous urticaria, gut microbiota, *Lachnospiraceae*, short-chain fatty acids

## Abstract

(1) Background: Chronic spontaneous urticaria (CSU) has been linked to the dysbiosis of the gut microbiota. Furthermore, various studies have highlighted the anti-inflammatory properties of short-chain fatty acids (SCFAs), whose production is primarily regulated by the gut microbiota. However, only a few studies have investigated the role of major SCFA producers, such as *Lachnospiraceae*, in skin inflammatory diseases. (2) Goal: This study aimed to compare the abundance of *Lachnospiraceae* between CSU patients and healthy controls (HCs). (3) Material and methods: In this case–control study, 16S rRNA sequencing was performed to compare the composition of the gut microbiome between 22 CSU patients and 23 HCs. (4) Results: Beta-diversity revealed significant clustering (*p* < 0.05) between the CSU patients and HCs. Alpha diversity in the CSU group was significantly decreased according to the Evenness index (*p* < 0.05). The linear discriminant analysis effect size (LEfSe) identified the significant depletion of the *Lachnospiraceae* family in CSU patients. (5) Conclusion: Our study revealed the dysbiosis of the gut microbiota in CSU patients, including decreased levels of *Lachnospiraceae* members, responsible for SCFA production, suggesting that SCFAs may contribute to immune dysfunction in the pathogenesis of CSU. We speculate that the modulation of SCFAs could serve as a prospective additional option in CSU treatment.

## 1. Introduction

Chronic spontaneous urticaria (CSU) is defined as the presence of urticaria, angioedema, or both, for a period of at least 6 weeks, without identifiable specific triggers [1]. It is characterized by daily or almost daily signs and symptoms or an intermittent course [1]. CSU affects about 1% of the general population and there has been a noticeable increase in the prevalence of this condition in recent years [2]. It is more frequently observed in young and middle-aged adults, affecting women two times more often than men [3]. However, children can be affected in the same proportion too [4]. The average duration of CSU is commonly around 5 years [5], although, in 10–25% of patients, it persists for more than 5 years [6]. The treatment of CSU is focused on “symptom control”, targeting mast cell mediators and activators such as histamine and autoantibodies. Second-generation non-sedating H1 antihistamines are recommended as the initial treatment (up to four-fold dose), followed by omalizumab as a second-line treatment. Other therapies include cyclosporin, glucocorticoids, and alternative treatments with limited evidence of efficacy [1]. Debilitating symptoms and the prolonged duration of the disease significantly impact individual’s quality of life; moreover, it represents large costs in the healthcare system [1,2].

To date, several theories regarding the pathogenesis of CSU have been proposed. However, none of them have been conclusively established. Research suggests that the autoimmune concept, which involves the response of IgE autoantibodies to aeroallergens or IgG antibodies to the patient’s own IgE or its high-affinity receptor-FcεRI, is the cause of a significant number of CSU cases [7]. This finding indicates that the immune system plays an important role in the development and persistence of this condition. However, other factors, such as the cellular defects theory, various infections, pseudoallergies, stress, coagulation, and vitamin D deficiency, have been proposed [8].

Recently, a growing number of studies have suggested a potential link between the development of CSU and the dysbiosis of the gut microbiota [9,10,11,12,13,14]. The human gut microbiota is a complex community of microorganisms, including bacteria, fungi, and viruses, that reside in the human gastrointestinal tract [14,15,16]. It plays a vital role in health maintenance, such as regulating the host immune system through the control of metabolism, the improvement of gut integrity, the prevention of pathogen propagation, and the modulation of components of both innate and adaptive immunity [15,16,17,18,19]. The overuse of antibiotics, poor hygiene, a diet characterized by a low fiber intake and high levels of fat and sugar, a sedentary lifestyle, pollution, and various toxins can disrupt the natural balance of gut bacteria, leading to dysbiosis [20,21].

Dysbiosis refers to an imbalance in the diversity and composition of microorganisms in the gut, which can lead to chronic inflammation, metabolic and immune dysfunction [20,21]. Alterations in the composition of gut microbiota have been linked to multiple infectious and non-infectious diseases such as inflammatory bowel diseases, diabetes mellitus, obesity, cardiovascular diseases, colorectal cancer, etc. [22,23]. Specifically, alterations in the gut microbiota composition and function have been observed in individuals with CSU compared to healthy individuals, suggesting the potential role of the gut microbiome in the pathogenesis of this condition [9,10,11,12,13,14]. While the exact mechanisms underlying this association are not yet fully understood, it is thought that the gut microbiota may impact the immune system and contribute to the development of chronic inflammation, which can trigger CSU symptoms [9,10,11,12,13,14,15].

One of the ways that the gut microbiome interacts with the host is through the production of metabolic products, such as short-chain fatty acids (SCFAs) [24]. SCFAs such as propionate, butyrate, and acetate are produced by gut bacteria through the degradation of non-digestible carbohydrates, vitamins, and immunomodulatory peptides [25]. Recent studies have reported the role of SCFAs in modulating the immune response in inflammatory skin diseases [24]. It has been proposed that SCFAs alleviate inflammation through the interaction and downregulation of components of both innate and adaptive immune systems [25,26]. The genera of the *Lachnospiraceae* family, part of the phylum Firmicutes, belongs to the core of the gut microbiota and are among the main producers of SCFAs [16]. The human gut has been found to harbor several dominant genera within the *Lachnospiraceae* family, including *Blautia*, *Coprococcus*, *Dorea*, *Lachnospira*, *Oribacterium*, *Roseburia*, and *L-Ruminococcus* [16]. Furthermore, their anti-inflammatory and immunomodulating effects on the human gut have been reported [27]. Therefore, there is growing interest in the research of *Lachnospiraceae*’s role in maintaining gut homeostasis. At present, there is an increasing number of studies reporting the role of SCFAs in chronic inflammatory diseases [24]. There have been a limited number of studies investigating the potential involvement of SCFA-producing bacteria in CSU [10,11,12]. However, it is important to note that certain studies have examined a combined cohort of both CSU patients and individuals with chronic inducible urticaria [11]. Liu et al. have reported a decrease in the relative abundance of SCFA producers in CSU patients and suggested *Subdoligranulum* and *Ruminococcus bromii* as potential markers for the diagnosis of CSU [10]. However, no research has specifically focused on *Lachnospiraceae* members, which are, along with *Ruminococceae*, the main producers of SCFAs. A study by Lu et al. observed a depletion of *Lachnospiraceae* in the gut microbiota of patients with alopecia areata [28]. Similarly, a study on the gut–skin axis in hidradenitis suppurativa showed differences in gut abundances of *Lachnospiraceae* between patients and healthy controls [29].

This study aimed to compare the composition and diversity of the gut microbiome between CSU and healthy controls (HCs) with an emphasis on identifying differences in the abundance of bacteria from the *Lachnospiraceae* family between the groups.

## 2. Materials and Methods

This case–control study was conducted at the Department of Dermatology and Venereology, Sestre milosrdnice University Hospital Centre in Zagreb, Croatia, between October 2020 and October 2021. A total of 45 participants were enrolled in the study, including 22 patients with CSU and 23 healthy individuals. The study was approved by the Research Ethics Committee of Sestre milosrdnice’s University Hospital Centre in Zagreb (Approval No 003-06/20-03/008, 2 April 2020). All participants provided written informed consent before participating in the study. The diagnosis of CSU was established according to the EEACI guidelines [1]. Participants with certain conditions that could affect gut microbiota composition were excluded from further investigation. The exclusion criteria were as follows: patients who had taken systemic antibiotics and commercial probiotics in the last 3 months, patients with inflammatory bowel diseases, diabetes, obesity, psychiatric diseases or malignancy, and patients who were pregnant. All 45 participants were instructed to provide gut samples at home and transfer them to the Department of Dermatology and Venereology within 7 days of entering the study. The participants followed the manufacturer’s instructions using the OM-200 OMNIgene GUT sample collection kit (DNA Genotek, Ottawa, ON, Canada). To collect the fecal samples, participants were instructed to use a spatula from the collection kit and transfer the samples into the provided tubes containing stabilizing liquid. After closing the tubes, participants were instructed to vigorously shake them for a minimum of 30 s to ensure the proper mixing of the feces with the stabilizing liquid. The stabilizing liquid rapidly homogenizes and stabilizes samples at the point of collection, ensuring that the microbiota profiles accurately represent the in vivo state. The manufacturer guarantees stabilized DNA at an ambient temperature for 60 days. All received samples were further stored at −80 °C until DNA extraction.

### 2.1. DNA Extraction and 16S rRNA Gene Sequencing

Microbial DNA from stool was isolated using a Zymo BIOMICS DNA Miniprep Kit #4300 (Zymo Research, Irvine, CA, USA), according to the manufacturer’s instructions [30]. Briefly, 250 mL of stool and 750 µL of Zymo BIOMICS Lysis Solution were put in ZR BashingBead Lysis Tubes and homogenized using a FastPrep FP120 Cell Disrupter (Thermo Electron Corporation, Milford, MA, USA) twice for 20 s, with a 30 s pause in between, at a speed of 4 [31]. All the steps followed were the same as those mentioned in the instruction manual, and at the end, the microbial DNA was eluted once in 50 µL of ZymoBIOMICS DNase/RNase-free water. The DNA concentration was measured on a Qubit 4 Fluorometer (Thermo Fisher Scientific, Waltham, MA, USA) using a Qubit 1× dsDNA High Sensitivity (HS) Assay Kit Q33230 (Thermo Fisher Scientific). DNA aliquots with a 5 ng/µL concentration were used for sequencing the library preparation. All extracted DNA samples were stored at −20 °C until further analysis.

The NGS libraries were prepared and sequenced following Illumina’s 16S metagenomic sequencing library preparation protocol (Part # 15,044,223 Rev. B; Illumina, San Diego, CA, USA) [32]. First, the 16S RNA gene amplicons were prepared using the primer pair targeting the V3–V4 hypervariable region of 16S rRNA genes [33]. Then, the PCR products were cleaned using MagSi-NGS PREP Plus (Magtivo, Nuth, The Netherlands), and Illumina dual-index barcodes were added to the amplicons using a Nextera XT Index Kit v2 set A (Illumina, (FC-131-2001)). The final pooled normalized library (4 nM), including controls, was diluted, denatured to 2 pM, and spiked with 15% PhiX (PhiX Control v3). A paired-end 300 bp sequencing run (600 cycles) was performed using the MiSeq platform (Illumina), using MiSeq Reagent Kit v3 chemicals (Illumina, (MS-102-3003)). All DNA quantity measurements were performed on a Qubit 4.0 Fluorometer (Thermo Fisher Scientific, Waltham, MA, USA) using the Qubit dsDNA HS Assay Kit (Thermo Fisher Scientifc).

### 2.2. Bioinformatics and Statistical Analysis

To analyze the 16S rRNA gene sequencing data, we utilized the QIIME2 bioinformatics platform [34]. The α-diversity of the bacterial populations, including the species richness and evenness, was calculated using several indices, including the Chao 1, Evenness, Faith’s phylogenetic diversity (PD), Observed OTUs, Shannon, and Simpson indices. Furthermore, we used Jaccard distance to determine the β-diversity between the two groups. Linear discriminant analysis (LDA) effect size (LEfSe) was performed to identify statistically significant differences in the relative abundance of gut microbial families and genera between the CSU patients and healthy individuals [35]. Only those LDA values greater than 2.5 and a *p*-value less than 0.05 were considered significantly enriched. 

The normality of continuous data was tested using the D’Agostino–Pearson test. Data with a non-normal distribution were presented with a median (range). Categorical data were presented as a number of samples (percentage). Mann–Whitney and Chi-squared tests were used to test the differences between two groups of continuous or categorical data, respectively. Statistical analyses were performed using Med Calc v20.218 (Med Calc Software, Ostend, Belgium) and R software v3.4.4 (R Foundation for Statistical Computing, Vienna, Austria). *p*-values < 0.05 were considered statistically significant.

## 3. Results

### 3.1. Characterization of Participants

The study included 45 participants, 22 patients with CSU, and 23 HCs. The observed groups did not exhibit significant differences in terms of gender and age. The basic information regarding the demographic and clinical data of the included participants is summarized in Table 1, Table 2 and Table 3. In terms of the appearance of urticaria, more than half of the CSU patients reported hives every day. The majority of patients (91%) reported taking second-generation non-sedating antihistamines daily.

About one-third of the CSU patients had associated angioedema or atopic disorders. Half of the patients had elevated TPO-Ab, and a significant proportion had elevated anti-Tg, which are both markers of autoimmune thyroid disease. Vitamin D deficiency was highly prevalent in CSU patients, with over two-thirds having hypovitaminosis.

### 3.2. Composition of Gut Microbiota

**Figure 1:**
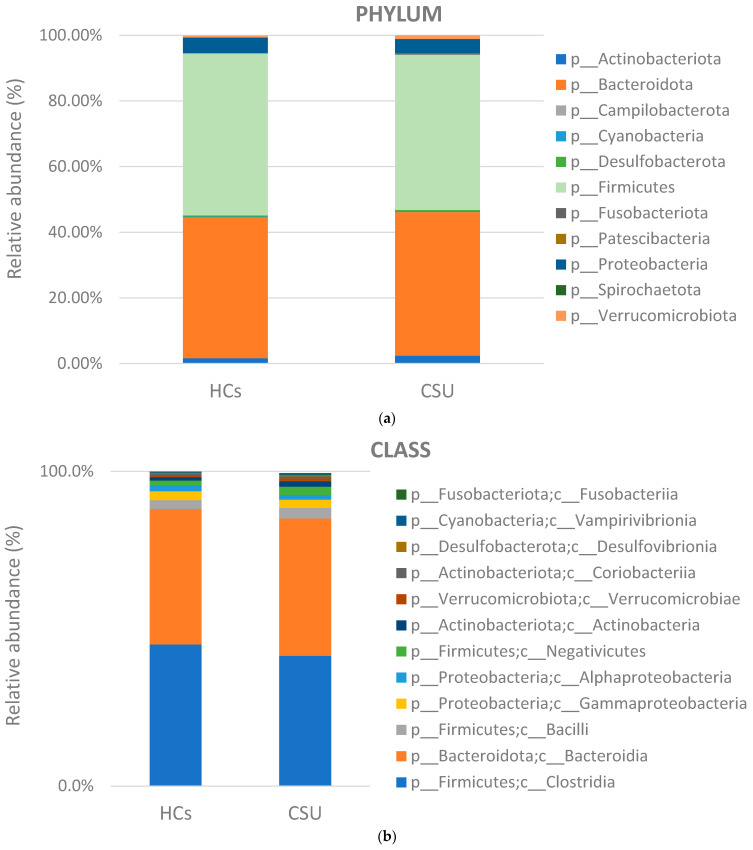

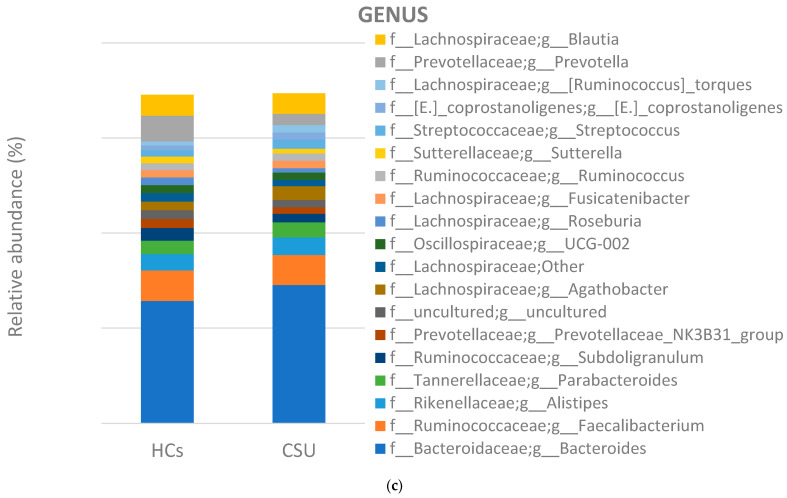
(**a**) The composition of the gut microbiota in CSU patients and HCs. Relative abundance at the phylum level. (**b**) Relative abundance at the class level. The most abundant taxa at the class level in both groups were *Clostridia* and *Bacteroidia. Clostridia* taxa were decreased in the CSU group. (**c**) Relative abundance at the genus level. The relative abundance of *Bacteroides*, *Blautia*, and *Bifidobacterium* was increased in the CSU group, while *Roseburia*, *Ruminococcus*, *Faecalibarium*, *Lachnospira*, *Prevotella*, *Coprococcus* and *Subdoligranulum* were decreased in the CSU group compared to the HCs.

**Figure 2:**
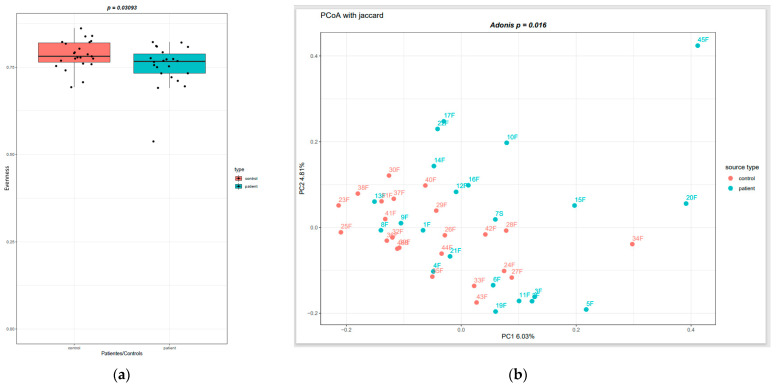
(**a**) Alpha diversity, measured by Evenness index. (**b**) Beta diversity, measured by Jaccard distance.

After quality control, a total of 3,633,362 sequences were obtained from the fecal samples of 45 subjects, resulting in an average of 80,741 sequences per sample. We examined the bacterial communities and relative abundance in the two groups at different taxonomic levels. At the phylum level, Firmicutes, Bacteroidetes, Proteobacteria, and Actinobacteria were found to be the dominant phyla in both CSU patients and healthy individuals. The most abundant taxes at different levels are shown in Figure 1a–c. The relative abundance of Firmicutes was increased in the HC group, while Bacteroidetes and Actinobacteria were more abundant in the CSU group. At the class level, the relative abundance of *Clostridia* was decreased in the CSU group. At the genus level, the relative abundances of *Bacteroides*, *Streptococcus*, *Agathobacter*, and *Bifidobacterium* were increased in the CSU group, while *Roseburia*, *Faecalibacterium*, *Ruminococcus*, *Lachnospira*, *Prevotella*, *Blautia*, *Coprococcus* and *Subdoligranulum* were decreased in the CSU group, compared to HCs. To evaluate the alterations in the gut microbial diversities between the CSU patients and HCs, we examined the α-diversity using the Chao 1, Evenness, Faith’s phylogenetic diversity (PD), Observed OTUs, Shannon and Simpson indices. Only the Evenness index showed a statistically significant difference (*p* < 0.05) between the two groups (Figure 2a), while the other metrics did not show a difference. The beta diversity, measured by the Jaccard distance between the CSU patients and the HCs, revealed the significant clustering (*p* < 0.05) of the CSU and HC groups. This finding supports the conclusion that there were notable differences in the gut microbiota composition between the two groups, as illustrated in Figure 2b.

**Figure 3:**
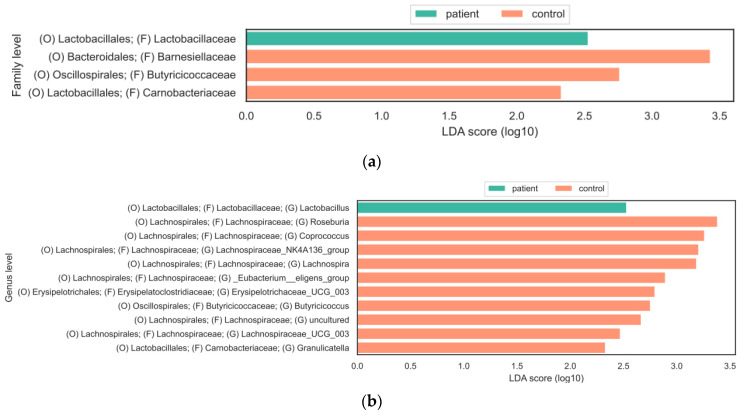
Linear discriminant analysis effect size (LEfSe) identified bacterial taxa that were significantly more abundant in the CSU group and HC group at the (**a**) family level and (**b**) genus level.

To explore the statistically significant differences in the relative abundance of gut bacteria between the two groups, LEfSe was performed. A bar plot was utilized to present the log10 LDA scores. At the family level, the results of the LEfSe analysis revealed that Lactobacillaceae were significantly more abundant in the gut microbiota of CSU patients compared to HCs. On the other hand, *Barnesiellaceae*, *Butyricicoccaceae*, and *Carnobacteriaceae* were more abundant in the control group compared to the patient group (Figure 3a). At the genus level, LEfSe identified a statistically significant increased abundance of bacteria from the *Lachnospiraceae* family in the HC group, including *Lachnospira*, *Roseburia*, *Ruminococcus*, *Coporcoccus*, and the *Eubacterium eligens group* (Figure 3b). On the other hand, the genus *Lactobacillus* was significantly increased in the CSU group.

## 4. Discussion

The gut microbiome has been the subject of intense research in recent years, and there is growing evidence that its composition and diversity are associated with various health and pathologic conditions, including CSU. In this study, we aimed to investigate the composition and diversity of the gut microbiome in CSU patients in comparison with HCs, using Illumina-based 16S rRNA gene sequencing. Our findings revealed significant alterations in the composition and diversity of the gut microbiome in CSU patients, compared to HCs. It is noteworthy that half of the included patients had elevated anti-TPO antibodies and one-third had anti-Tg antibodies, which is in line with the concept of CSU as an immune-mediated disease. 

To assess the differences in the diversity of the gut microbiome, we used different indices for alpha and beta diversity. Alpha diversity refers to the diversity of bacterial species within a single individual. Beta diversity, on the other hand, refers to the diversity of bacterial species between individuals. Analysis of beta diversity showed that the microbiota composition differed significantly between the groups, as was shown in the principal co-ordinates analysis (PCoA). This clustering is in line with previously published papers on gut microbiota in CSU [10,12,13]. We observed a significant decrease in the alpha diversity of the gut microbiota in the CSU group, according to the Evenness index, while other indices did not show statistically significant differences. We speculate that this bias may be due to a large range of ages within the group, differences in dietary habits, and demographic data. The CSU and HCs groups were mainly composed of Firmicutes, Bacteroidetes, Actinobacteria, and Proteobacteria, consistent with similar studies. At the phylum level, the relative abundance of Bacteroidetes and Actinobacteriota was slightly increased in the CSU group, while phylum Firmicutes was decreased, which has also been reported in studies by Wang et al. [11] and Liu et al. [10]. At the class level, *Clostridia* was relatively more abundant in the HC group, while the relative abundance of *Bacteroides* was slightly more increased in the CSU group. Genera of the *Lachnospiraceae* family, as well as *Subdoligranulum*, from the *Ruminococcaceae* family, were relatively decreased in the CSU group. Of note, we found decreased *Prevotella* spp. in the CSU group compared to healthy individuals, which is in concordance with a study by Lu et al. [9]. Emerging studies have linked the increased abundance of *Prevotella* with metabolic changes in the gut microbiota, resulting in chronic inflammation [36,37]. In contrast, Hilty et al. reported a reduced abundance of *Prevotella* in the lung microbiota of asthma patients and patients with chronic obstructive pulmonary disease [38]. Furthermore, a study by Chen et al. investigated the relationship between the composition of the gut microbiota and its ability to utilize dietary fiber. The results showed that the *Prevotella*-dominated group had a higher fiber-utilizing capacity compared to the *Bacteroides*-dominated group. The authors observed that a high-fiber diet increased the abundance of *Prevotella* and the expression of fiber-utilizing genes [39]. Therefore, the role of *Prevotella* in disease development requires further investigation.

Furthermore, we used LefSe analysis to identify specific bacterial taxa that were significantly more abundant in either the CSU group or the healthy controls. We found significant changes in the bacterial composition at the family and genus levels. Interestingly we found statistically significant increased levels of *Lactobacillaceae* at the family level and *Lactobacillus* at the genus level in the CSU group. On the other hand, *Coprococcus*, *Lachnospira*, *Roseburia*, *Lachnospiraceae NK4A136*, *Lachnospiraceae_UCG_003*, *Eubacterium eligens*, *Erysipelotrichaceae_UCG_003*, and *Granulicatella* were significantly more abundant in the healthy controls. An increased abundance of *Lactobacillaales* in CSU patients has also been reported in a study by Lu et al. [9], while Wang et al. reported an increased genus of *Lactobacillus* in the CSU group [12]. The potential role of *Lactobacillus* in the prevention and treatment of allergic diseases has been observed [40]. However, the results of clinical trials and observational studies examining the efficacy of *Lactobacillus* administration in reducing the risk of allergic diseases have been inconsistent. Some studies have reported no significant effect of *Lactobacillus* supplementation on the incidence of eczema, asthma, or cow’s milk allergy [41,42]. It is important to note that the inconsistencies in the findings of these studies may be due to differences in the study design. Despite these mixed findings, the potential beneficial role of *Lactobacillus* in preventing or treating allergic diseases should not be overlooked. Further research is needed to better understand the mechanisms by which *Lactobacillus* may exert its beneficial effects on the host.

*Coprococcus, Lachnospira, Roseburia, Lachnospiraceae NK4A136*, *Lachnospiraceae_UCG_003*, and *Eubacterium eligens* are members of the *Lachnospiraceae* family, which belongs to the clostridial cluster XIVa of the phylum Firmicutes [16,43]. The *Lachnospiraceae* family comprises strictly anaerobic bacteria, which belong to the core of gut microbiota and are one of the most abundant bacteria in the human gut. About 10% of all microbes present in the human gut are members of the *Lachnospiraceae* family [44]. *Lachnospiraceae* start to inhabit the intestinal lumen from birth, and their abundance increases during the host’s life [16], so they have been detected in individuals of all age groups, from infants to elderly people [27,45,46].

Due to its high abundance and lifelong association with the host, studies have emphasized the role of the *Lachnospiraceae* family in maintaining gut homeostasis and the overall health of the host [27]. However, a reduction in *Lachnospiraceae* within the gut microbiota has been associated with a range of conditions, including allergies, inflammatory bowel disease, and metabolic disorders [16,47].

Along with *Lactobacillaceae* and *Ruminococcaceae*, *Lachnospiraceae* are the main bacteria in charge of producing SCFAs [48,49,50]. 

SCFAs are a group of fatty acids with fewer than six carbon atoms that are produced by the bacterial fermentation of non-digestible carbohydrates in the gut, such as resistant starch, polysaccharide plant cell walls, soluble oligosaccharides, etc. [21,51,52]. The main SCFAs produced by gut bacteria are acetate, propionate, and butyrate, with butyrate being the most abundant. The main butyrate-producing bacteria in the human gut belong to the phylum Firmicutes, including *Faecalibacterium prausnitzii*, *Subdoligranulum*, and *Clostridium leptum*, which belong to the families *Ruminococcaceae*, *Eubacterium rectale* and *Roseburia* spp. from the family *Lachnospireacae* [53,54]. Our study reported decreased genus levels of all the above-mentioned bacteria in CSU patients compared to healthy individuals. 

Following fermentation, the intestinal lumen contains millimolar concentrations of short-chain fatty acids (SCFAs), which are absorbed by the epithelium through both active and passive transport mechanisms. The SCFAs are subsequently transported to distant organs and tissues via peripheral circulation [55,56]. SCFAs play various roles in host metabolism and the immune system. They have an impact on the metabolism of lipids and glucose. Furthermore, they are important for the integrity and protection of the intestinal barrier, with butyrate being the dominant SCFA in charge of this function. Similar beneficial effects in improving the intestinal barrier were found after the administration of the probiotic *Butyrococcus pullicaecorum* to patients with Crohn’s disease [57]. Our study also showed a lower abundance of *Butyrococcus* at the genus level in CSU patients. 

SCFAs can mediate their effects via multiple mechanisms, including binding to G-protein coupled receptors (GPCRs). GPCRs are the largest family of receptors with seven-transmembrane domains. These receptors play a crucial role in regulating various important cellular functions, including cell proliferation and survival, metabolism, and neuronal signal transmission [58].

SCFAs bind to GPCRs such as GPR41, GPR41, and GPR109A. These receptors are expressed in various cells, including neutrophils, leukocytes, skin cells, etc. [59,60]. SCFA-mediated GPCRs initiate signaling pathways that play a vital role in regulating various cellular responses, immune functions, and inflammatory processes; they, therefore, have an important role in regulating the inflammatory response of the host [61]. Furthermore, the anti-inflammatory effects of SCFAs, especially butyrate, have been attributed to their ability to inhibit histone deacetylase (HDAC) activity. This leads to a decrease in the production of pro-inflammatory mediators such as TNF-α, IL-6, and IL-12 while increasing the production of anti-inflammatory mediators such as IL-10. Additionally, butyrate has been found to promote *FOXP3* expression in naïve CD4+ T-cells and facilitate their differentiation into Tregs by inhibiting HDACs. Similarly, acetate, propionate, and butyrate have been shown to modulate the immune response of dendritic cells, macrophages, and Treg cells by inhibiting HDACs [62,63]. Treg cells can suppress pro-inflammatory cytokines mediated by various T-cells, including Th2 cells, and can induce the secretion of anti-inflammatory mediators, thereby reducing inflammation [14]. Reduced Treg cells have been observed in patients with CSU [64,65,66]. GPCRs are also expressed in mast cells, the key cells in the development of urticaria. SCFAs can bind to receptors on mast cells, resulting in the inhibition of mast cell activation and inflammatory response [67,68]. Moreover, studies have shown that not only receptors such as GPR41, GPR43, or peroxisome proliferator-activated receptors (PPARs) are responsible for mast cell inhibition, but that HDAC also independently inhibits their maturation and degranulation [69]. Liu et al. suggest that decreased levels of *Subdoligranulum* and *Ruminococcus bromii* can promote mast cell activation and degranulation, leading to the development of hives and itching in CSU patients [10]. Recent studies have shown that CSU patients have lower levels of fecal isobutyrate and serum butyrate compared to healthy individuals [11,12]. The *Roseburia/Eubacterium* group and *Lachnospiraceae NK4A136* group produce a high amount of butyrate, which is involved in controlling gut inflammatory processes and immune system maturation [70]. Our study reported a reduction in all the above-mentioned bacterial groups in CSU patients, which is consistent with emerging studies suggesting that a reduction in the number of SCFA-producing bacteria may contribute to the development of CSU. In addition to SCFAs, several other mechanisms underlying the impact of *Lachnospiraceae* bacteria on the gut microbiota have been proposed. Certain *Lachnospiraceae* species have been found to utilize mucin, a glycoprotein from the protective mucus layer of the gut. By breaking down mucin, *Lachnospiraceae* can promote the turnover of the mucus layer, which is important for protecting the gut from pathogens [16]. *Lachnospiraceae* can engage in cross-feeding interactions with other members of the gut microbiota by consuming the byproducts of other bacteria and producing metabolites that benefit gut stability and functionality. They also can promote Treg cells and downregulate pro-inflammatory cytokines and Toll-like receptor 4 (TLR4) [16,45]. It is important to note that the specific effects of *Lachnospiraceae* bacteria on the gut microbiota may vary depending on the species and strains within this family. Additionally, individual variations in the gut microbial composition and host factors can influence the impact of *Lachnospiraceae* on gut health. The gut microbiota is influenced by various factors such as infections, antibiotics, age, lifestyle, diet, and environmental exposures [20]. Moreover, changes in the composition of the gut microbiota have been associated with many diseases and cancers [71]. Our findings suggest that alterations in the gut microbiota may contribute to the pathogenesis of CSU. Furthermore, we found a lower abundance of *Lachnospiraceae*, a core family of the gut microbiota known for producing SCFAs, in CSU subjects. This study has several limitations: a small sample size which may not be large enough for strong conclusions; the study was conducted in a single center, which may limit the generalizability of the findings; and while the study excluded participants with certain medical conditions and medication use, it did not report information regarding the dietary habits of the participants, which can have a significant impact on the gut microbiome. Considering the limitations encountered in our study, we plan to expand our sample size and classify participants into groups based on age and dietary habits. By undertaking these measures, our objective is to obtain a more comprehensive representation of the diverse microbial profiles, thereby augmenting the validity of our findings.

The gut microbiome and its interaction with the host are critical for maintaining the health and homeostasis of the organism. SCFAs have been shown to play an important role in the regulation of metabolism and the integrity of the intestinal barrier, as well as in the modulation of the immune response. Moreover, SCFAs have shown promising results in reducing inflammation in inflammatory skin diseases. Thus, the reduced abundance of *Lachnospiraceae*, SCFA-producing bacteria, may contribute to the development of CSU. Herein, we speculate that the modulation of SCFAs and SCFA-producing organisms could be a potential additional option in the treatment of CSU.

These findings provide important insights into the potential role of gut microbiota in the pathogenesis of CSU and highlight the need for continued research in this area.

## Figures and Tables

**Table 1:** Characteristics of the CSU patients and healthy controls.

Variable	CSU Patients (*N* = 22)	Healthy Controls (*N* = 23)	*p*-Value
Median age, years (range)	42 (20–73)	40 (19–74)	0.928
Sex, *n* (%)			
Male	6 (27.3%)	7 (30.4%)	0.817
Female	16 (72.7%)	16 (69.6%)	

**Table 2:** Clinical parameters of CSU patients.

Variable	CSU Patients (*N* = 22)
Duration of symptoms	
6 weeks–5 months, *n* (%)	15 (68%)
6 months–12 months, *n* (%)	7 (32%)
Appearance of urticaria	
everyday, *n* (%)	13 (59%)
2–4 times a week, *n* (%)	8 (36%)
once a week, *n* (%)	1 (5%)
Taking non-sedating antihistamines *n* (%)	20 (91%)
1 tablet daily, *n* (%)	5 (23%)
2 tablets daily, *n* (%)	8 (36%)
3 tablets daily, *n* (%)	3 (14%)
4 tablets daily, *n* (%)	4 (18%)

**Table 3:** Laboratory parameters of CSU patients.

Variable	CSU Patients (*N* = 22)
Associated angioedema, *n* (%)	7 (32%)
Associated atopic disorders, *n* (%)	7 (32%)
Elevated Anti-Tg, *n* (%)	6 (27%)
Elevated TPO-Ab, *n* (%)	11 (50%)
Vitamin D deficiency, *n* (%)	15(68%)
Elevated IgE, *n* (%)	9 (41%)

Abbreviation: Anti-Tg—anti-thyroglobulin; TgTPO-Ab—thyroid peroxidase antibodies.

## Data Availability

The corresponding author can be contacted for access to the data presented in this study as they are not publicly available due to the inclusion of the personal information of the study participants.
